# In Vivo MR Imaging of Pulmonary Perfusion and Gas Exchange in Rats via Continuous Extracorporeal Infusion of Hyperpolarized ^129^Xe

**DOI:** 10.1371/journal.pone.0031306

**Published:** 2012-02-21

**Authors:** Zackary I. Cleveland, Harald E. Möller, Laurence W. Hedlund, John C. Nouls, Matthew S. Freeman, Yi Qi, Bastiaan Driehuys

**Affiliations:** 1 Department of Radiology, Center for In Vivo Microscopy, Duke University Medical Center, Durham, North Carolina, United States of America; 2 Max Planck Institute for Human Cognitive and Brain Sciences, Leipzig, Germany; 3 Graduate Program in Medical Physics, Duke University, Durham, North Carolina, United States of America; University of Giessen Lung Center, Germany

## Abstract

**Background:**

Hyperpolarized (HP) ^129^Xe magnetic resonance imaging (MRI) permits high resolution, regional visualization of pulmonary ventilation. Additionally, its reasonably high solubility (>10%) and large chemical shift range (>200 ppm) in tissues allow HP ^129^Xe to serve as a regional probe of pulmonary perfusion and gas transport, when introduced directly into the vasculature. In earlier work, vascular delivery was accomplished in rats by first dissolving HP ^129^Xe in a biologically compatible carrier solution, injecting the solution into the vasculature, and then detecting HP ^129^Xe as it emerged into the alveolar airspaces. Although easily implemented, this approach was constrained by the tolerable injection volume and the duration of the HP ^129^Xe signal.

**Methods and Principal Findings:**

Here, we overcome the volume and temporal constraints imposed by injection, by using hydrophobic, microporous, gas-exchange membranes to directly and continuously infuse ^129^Xe into the arterial blood of live rats with an extracorporeal (EC) circuit. The resulting gas-phase ^129^Xe signal is sufficient to generate diffusive gas exchange- and pulmonary perfusion-dependent, 3D MR images with a nominal resolution of 2×2×2 mm^3^. We also show that the ^129^Xe signal dynamics during EC infusion are well described by an analytical model that incorporates both mass transport into the blood and longitudinal relaxation.

**Conclusions:**

Extracorporeal infusion of HP ^129^Xe enables rapid, 3D MR imaging of rat lungs and, when combined with ventilation imaging, will permit spatially resolved studies of the ventilation-perfusion ratio in small animals. Moreover, EC infusion should allow ^129^Xe to be delivered elsewhere in the body and make possible functional and molecular imaging approaches that are currently not feasible using inhaled HP ^129^Xe.

## Introduction

The hyperpolarized (HP) noble gases ^3^He and ^129^Xe [1], [2] have emerged as promising contrast agents for magnetic resonance imaging (MRI) of pulmonary structure and function. In particular, HP ^3^He MRI has been used to evaluate pathological changes in pulmonary microstructure and ventilation patterns. HP ^129^Xe MRI, despite its intrinsically lower signal intensity, has recently yielded comparable information to that obtained from HP ^3^He in both small animals [3] and humans [4], [5]. More interestingly, xenon is soluble in tissues [6], and ^129^Xe possesses a large (>200 ppm) chemical shift range, making it a powerful probe of the local chemical environment within biological systems [7], [8], [9], and these properties can be exploited to directly image gas uptake in the lungs of rodents [10] and humans [11], [12].

Additionally, the solubility of HP ^129^Xe enables functional imaging studies that do not depend on inhaling the gas and, thus, are not necessarily limited to the ventilated portions of the lungs. For instance, HP ^129^Xe can first be dissolved in a biologically compatible carrier and then injected directly into the vasculature [13]. When dissolved in saline, injected HP ^129^Xe rapidly passes from the pulmonary capillaries into the alveolar airspaces [14] due to a combination of pulmonary perfusion and diffusive gas exchange [15], and its resonance frequency is shifted by ∼200 ppm, allowing ^129^Xe residing in the airspaces to be selectively detected and imaged. That is, once delivered to the vasculature, HP ^129^Xe travels the same physical path as CO_2_, making it a unique spatially and temporally resolved probe of gas elimination.

In earlier work with HP ^129^Xe saturated saline [14], these properties enabled MR imaging of both normal and impaired gas exchange in rats. Unfortunately, saline delivery is constrained by the tolerable injection volume and provides HP ^129^Xe signal for only ∼20 seconds. To extend the lifetime of the HP ^129^Xe signal and eliminate the need for carrier solutions, we recently developed a method that allows ^129^Xe to be infused directly into flowing aqueous solutions [16] by using hydrophobic, gas-exchange membranes [17], [18], [19]. Here, as an initial *in vivo* demonstration, we show that membrane-based delivery enables extracorporeal (EC) infusion of HP ^129^Xe into the blood of live rats. Using ∼300 ml of HP ^129^Xe gas, EC infusion can provide continuous signal to the lungs for more than 30 minutes. Furthermore, these long-term signal dynamics are well described by an analytical model that incorporates both mass transport across the gas-exchange membranes and the effects of longitudinal relaxation. Finally, we also show that the HP ^129^Xe signal intensity generated during EC infusion of rats is sufficient to generate 3D MR images of the lungs that reflect both diffusive gas exchange and pulmonary perfusion.

## Materials and Methods

### Method Overview

HP ^129^Xe was infused into blood using a Liqui-Cel MicroModule™ (Membrana, Charlotte, NC), which is similar to the gas-exchange modules used in extracorporeal blood oxygenation [20] and contains hollow fibers (Celgard® X50, Membrana) that consist of microporous (0.04-µm pores), polypropylene membranes. Within the module, blood was located on the exterior of these hollow fibers and was prevented from wetting the microscopic pores, and thus flooding the interior of the fibers, by the hydrophobicity of the polypropylene membranes. In contrast, the gaseous HP ^129^Xe inside the fibers passively diffused through the pores and into the blood [16], [17].

**Figure 1:**
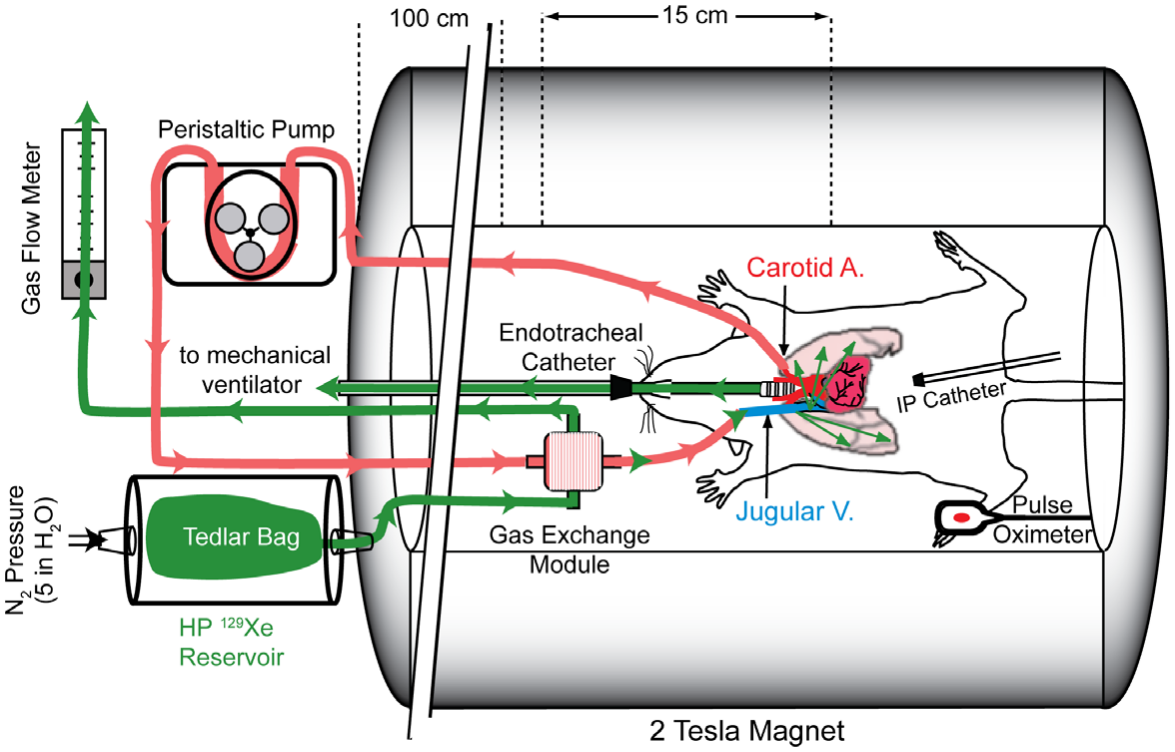
Schematic depicting the EC infusion of HP ^129^Xe into the blood of a live rat. HP ^129^Xe gas flows from a Tedlar bag located within the pressurized cylinder into the gas-exchange module where it is infused into arterial blood. Simultaneously, blood is withdrawn from the carotid artery using a variable speed peristaltic pump, passed through the gas-exchange module, and returned to the animal via the jugular vein. HP ^129^Xe is then transported by perfusion to the lung parenchyma, where it diffuses from the pulmonary capillaries into the adjacent alveolar airspaces.

For gases with moderate solubilities such as xenon (Ostwald solubility, *L* = 0.09 in blood plasma and 0.27 in red blood cells [6]), gas concentration in the fibers necessarily exceeds the concentration dissolved in the blood. Thus, by constantly supplying excess xenon, steady-state mass transport across the membrane can be made essentially independent of gas flow [21], and gas flows as low as 5 ml/min are sufficient to enable continuous HP ^129^Xe delivery [16]. Excess HP ^129^Xe that was not dissolved by the blood was allowed to pass through the module and out of the magnet (see Fig. 1). In doing so, gas pressure within the hollow fibers was maintained below that of blood flowing outside the membranes, and bubble formation was avoided [16]. Blood was provided to the module by withdrawing it from the carotid artery. HP ^129^Xe-infused blood from the exchange module was returned to the animal via the jugular vein, carried by the venous blood to the heart, and then, via the pulmonary arteries, to the lung parenchyma. After reaching the pulmonary gas-exchange region, ^129^Xe diffused from the pulmonary capillaries into the alveolar airspaces.

### HP ^129^Xe Accumulation and Infusion

Isotopically enriched xenon (83% ^129^Xe, Spectra Gases Inc., Alpha, NJ) was hyperpolarized by spin exchange optical pumping [1], [2] using a prototype commercial polarizer (model 9800, MITI, Durham, NC) that employs a continuous flow of 1% xenon, 89% helium, and 10% N_2_ passing through a rubidium vapor-containing optical cell. After exiting the cell, HP ^129^Xe was cryogenically extracted from the buffer gases using liquid nitrogen [22] with final polarizations of ∼10%. Following accumulation, HP ^129^Xe was infused into blood flowing through the EC circuit (see Fig. 1) in a manner similar to that described by Cleveland *et al.* [16]. Briefly, up to 300 ml of polarized xenon was thawed into 350-ml Tedlar bags (Jensen Inert Products, Coral Springs, FL) located inside a Plexiglas cylinder that had been aligned with the magnet bore to minimize transverse magnetic field gradient-induced relaxation [23]. The bag was then pressurized to ∼5 in. H_2_O above ambient pressure with N_2_ gas. HP ^129^Xe flow was initiated by opening a plastic stopcock and was infused directly into the blood using the gas-exchange module. Gas flow was held constant using a direct reading flow meter (Cole-Parmer, Vernon Hills, IL) located inline beyond the exchange module.

Note, the liquid volume within the module included a relatively large (∼0.6 ml) region that contained no gas-exchange membranes. To reduce dissolved longitudinal relaxation within this region [16], this empty volume was filled with 0.5-mm glass beads (BioSpec Products Inc., Bartlesville, OK) that had been silicone-treated (Surfasil, Thermo Scientific, Bellefonte, PA) as described by Stupic *et al.* [24]. This modification was employed to provide increased signal intensity for signal dynamics experiments performed during a single breath and during imaging experiments.

### Animal Preparation

Animals were prepared in accordance with procedures approved by the Duke University Institutional Animal Care and Use Committee. Duke University is fully accredited by the Association for Assessment and Accreditation of Laboratory Animal Care International (AAALAC) and has current PHS-NIH Animal Welfare Assurance (A3195-01). Two groups of male, Sprague Dawley rats (Charles River, Wilmington, MA) weighing 290–420 g were used in this study. The first group of rats (N = 10) were used for optimizing the surgical procedures and for determining appropriate experimental parameters (e.g., EC flow rate, heparin dosage, etc.) in bench-top experiments that did not involve MR imaging or spectroscopy, and 6 rats were used for MR experiments. Animals in both groups were initially anesthetized by intraperitoneal (IP) injection of 50 mg/kg sodium pentobarbital (Nembutal, Lundbeck, Inc., Deerfield, IL) and 1.0 mg/kg butorphanol tartrate (Torbugesic, Fort Dodge Animal Health, Inc., Fort Dodge, IA). Anesthesia was maintained with periodic injections of Nembutal (20 mg/kg) administered via an IP catheter. Following experiments, all animals were euthanized by pentobarbital overdose.

For MR experiments, rats were positioned supine in the radio frequency (RF) coil. Body temperature was monitored via a rectal thermistor and maintained at ∼37°C by warm air flowing through the magnet bore. Rats were intubated by tracheostomy with a 16-gauge catheter (Abbocath-T, Hospira Venisystems, Lake Forest, IL) to provide an airtight seal for mechanical ventilation. Animals were ventilated at a rate of 1 breath/s on an HP-gas compatible, constant-volume, mechanical ventilator [25] with a tidal volume of 1.0 ml/100 g body mass. A breathing gas mixture of 65% N_2_ and 35% O_2_ was used to maintain blood oxygen saturation during extracorporeal circulation (see Results and Discussion: Physiological Effects of HP ^129^Xe EC Infusion).

During ventilation imaging, the O_2_ concentration in the breathing mixture was kept constant, but N_2_ gas was replaced with an equal volume of HP ^129^Xe. Each breath comprised a 250-ms inhalation, 20-ms breath-hold, and a 730-ms period of passive exhalation. For EC infusion spectroscopy experiments, the breath-hold duration was 200 ms, and the passive exhalation period was 550 ms. Airway pressure was monitored by a pressure transducer attached to the ventilation tube. Heart rate and blood oxygen saturation were measured by an MRI-compatible, small animal pulse oximeter (MouseOx, STARR Life Sciences Corp., Oakmont, PA) attached to the hind foot. The pulsed oximeter also reported pulse distension, which was used as a relative measure of peripheral, arterial pressure.

Prior to EC infusion, animals were injected in the right jugular vein with heparin (<420 IU/kg/hr) to prevent blood clotting. Blood was removed from the animal through a PE-50 catheter placed in the left carotid artery with the tip positioned near the aortic arch. Beyond the catheter entry point, the carotid artery was ligated to prevent bleeding. Blood was returned to the body via a 3-French catheter placed in the right jugular vein with the tip located near the right atrium. EC blood flow was achieved between the carotid artery and jugular vein by passing the blood through the closed circuit with a three-roller peristaltic pump (MasterFlex, Cole-Parmer). Prior to initiating EC blood flow, the circuit, having a total volume including the gas-exchange module of ∼3.5 ml, was primed with warm (∼37°C), heparinized (0.1% heparine) hetastarch solution (Hextend, Hospira Inc., Lake Forest, IL). Note, hetastarch was chosen as a priming fluid, because it is non-crystalloid solution and therefore expected to decrease the likelihood of pulmonary edema during extracorporeal circulation compared to crystalloid solutions such as saline [26]. While the animal was in the magnet, the EC circuit was warmed using a water jacket heated with a recirculating bath (PolyScience Model 210, Niles, IL). Blood volume, and thus pulse distention, was maintained with periodic injections (1–2 ml/hr) of hetastarch delivered through the jugular vein.

### MR Spectroscopy and Imaging

Spectra and images were acquired using a 2.0-T horizontal, 30-cm bore magnet (Oxford Instruments, Oxford, UK) equipped with 180 mT/m shielded gradients and controlled by a GE EXCITE 12.0 console (GE Healthcare, Milwaukee, WI). The scanner was interfaced to a custom-built, 23.66-MHz, quadrature birdcage coil (8-cm long, 7-cm diameter) by an integrated transmit/receive switch with 31-dB gain preamplifier (Nova Medical, Wilmington, MA) and was made to operate at 23.66 MHz instead of its intrinsic 63.86-MHz frequency using an up-down converter (Cummings Electronics Labs, North Andover, MA).

Prior to imaging, rats were ventilated with the 1% ^129^Xe mixture flowing from the polarizer [27], and this direct-flow HP ^129^Xe was used to localize the animal, set the RF frequency, calibrate the RF flip angle, and perform *in vivo* shimming. High-resolution ventilation images were acquired using cryogenically concentrated HP ^129^Xe (i.e., 100% xenon) with a field of view (FOV) of 64 mm in all dimensions (without slice selection) using a 3D radial sequence that employed a pseudo-random view ordering [28]. The acquisition comprised 6435 radial views obtained over multiple breaths at end-expiration and at a rate of 40 views/breath. HP ^129^Xe was selectively excited using 1.2 ms, 3-lobe sinc pulses and a variable flip angle scheme [29] that provided uniform signal for each view and consumed all of the available magnetization. Additional imaging parameters included: bandwidth (BW) = 8 kHz, repetition time (TR) = 10 ms, and echo time (TE) = 0.9 ms. Radial images were reconstructed with a nominal resolution of 1×1×2 mm^3^ using a non-uniform fast Fourier transform (NUFFT) algorithm [30] that employed a least squares optimized kernel to interpolate the nonuniform, radial k-space data onto a 64×64×32 matrix prior to Fourier transform. Image registration was performed using routines written in MATLAB (MathWorks, Inc., Natick, MA).

Gas-phase, EC infusion images were generated using the same radial sequence (views = 4801, FOV = 64 mm, matrix = 32×32×32, resolution = 2×2×2 mm^3^, and TE = 0.9 ms) and reconstruction algorithm. For these images, radial views (TR = 180 ms, constant flip angle = 40°) were also acquired at end-expiration, but at a reduced rate of 4 views/breath to allow for increased signal intensity (see Results and Discussion: Gas-Phase Signal Dynamics within a Breath). Dissolved-phase, EC infusion images were acquired with the same parameters as the gas-phase infusion images except that BW was increased to 15.6 kHz to compensate for the faster dissolved-phase T_2_^*^ (∼2 ms versus ∼20 ms in the gas-phase as determined by ^129^Xe NMR spectroscopy). Image signal-to-noise ratio (SNR) measurements were made by selecting regions of interest in ImageJ (National Institutes of Health, Bethesda, MD, http://rsb.info.nih.gov/ij/).

Spectra were obtained by selectively exciting gas-phase ^129^Xe and processed using HiRes Version 1.6 (Hatch Center for MR Research, Columbia University, New York, NY) and routines written in MATLAB. Magnetization dynamics simulations were also performed in MATLAB.

### Mathematical Model of Magnetization Dynamics

Assuming that gas exchange is instantaneous on the timescale of the data acquisition, the HP ^129^Xe magnetization arriving in the alveolar spaces, 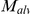, during vascular delivery can be expressed as [14]

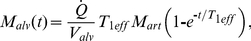
(1)where 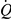 is pulmonary perfusion, 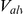 is alveolar volume, and 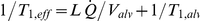 is the effective longitudinal relaxation rate, in which *L* is the Ostwald solubility of xenon and 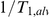 is true alveolar relaxation rate. The source of alveolar magnetization in Eq. 1 is the ^129^Xe magnetization dissolved within the pulmonary artery, *M_art_*.

The dissolved ^129^Xe magnetization reaching the detection region during membrane infusion, in this case *M_art_*, can be expressed as [16]

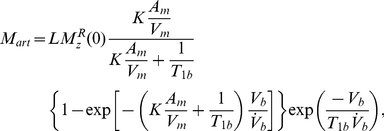
(2)where *A_m_* is the surface area of the gas-exchange membranes, *V_m_* is the liquid or “empty” volume within the module, 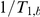 is the relaxation rate of ^129^Xe in blood, 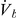 is the blood flow in the EC circuit, *V_b_* is the blood volume between the module and the pulmonary artery, 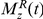 is the magnetization arriving at the gas-liquid interface in the module at time *t*, and *K* is the mass transfer coefficient for xenon into blood. (Note, gas-phase mass transport due to diffusion through the pores of the hollow-fiber membrane is essentially instantaneous on the timescale of overall mass transport and can be neglected [31].) Literature values for the parameters used in this model are provided in Table 1.

**Table 1:** Symbols and Literature Values Used in the Signal Dynamics Model.^A^

Symbol	Meaning	Value	Ref.
a,b,c	Empirical mass transport constants	*a* = 0.8, *b* = 0.59, *c* = 0.33	[38]
A_m_	Gas-exchange surface area of the module	0.010 m^2^	B
A_R_	Surface area of the Tedlar reservoir	328 cm^2^	C
D	Diffusion constant of dissolved xenon	1.51×10^−9^ m^2^ s^−1^ D	[54]
d_e_	Equivalent diameter of the membrane fibers	d_e_ = εd_0_/(1−ε)	[38]
d_o_	Outer diameter of the membrane fibers	3×10^−4^ m	B
ε	Void fraction of the gas-exchange module	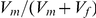	
κ	Surface relaxivity of Tedlar for ^129^Xe	0.392±0.008 cm h^−1^	[34]
Κ	Mass transport coefficient of a power law fluid	Eq. 3	[32]
L	Ostwald solubility of xenon in blood	0.165^E^	[6]
m	Flow consistency index	0.01396 Pa s^F^	[33]
n	Flow behavior index	0.63^F^	[33]
M_art_	Dissolved arterial ^129^Xe magnetization	Eq. 2	
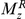	^129^Xe magnetization at the gas-liquid interface	Eq. 6	
ρ	Density of blood	1.051 kg m^−3^ E	[55]
Re_P_	Reynolds number of a power law fluid	Eq. 4	[32]
Sc_P_	Schmidt number of a power law fluid	Eq. 5	[32]
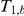	^129^Xe longitudinal relaxation time in blood	4.0 s^E^	[56]
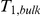	Bulk ^129^Xe longitudinal relaxation time in the xenon gas reservoir	2.55±0.22 h	[34]
u	Average blood velocity within the module	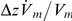	
V_b_	Blood transfer volume after the module	5.0×10^−7^ m^3^	
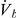	Blood flow through the EC circuit		
V_f_	Total volume of hollow fibers	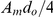	
V_g_	Gas volume in the reservoir	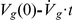	
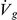	Gas flow from the reservoir		
V_m_	Empty volume (liquid volume in the module)	2.7×10^−6^ m^3^	B
Δz	Module length in the direction of liquid flow	0.020 m	B

A The hematocrit for adult, male Sprague-Dawley rats is ∼0.40 [57]. Here we assume 0.35 to account for dilution by priming fluid and hetastarch injections.

B From Membrana/Celgard technical literature.

C Geometric surface area.

D Has not been reported in whole blood. Stated value is in human blood plasma at 37°C.

E Human blood.

F Bovine blood.

Before the mass transport coefficient can be calculated, the rheological properties of blood passing through the module must be considered. Specifically, whole blood is a shear thinning, non-Newtonian fluid, meaning that the value of 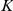 during EC infusion will depend on blood flow inside the exchange module. Fortunately, for shear thinning fluids, including blood, mass transfer using hollow-fiber gas-exchange membranes can be well described if liquid is modeled as a power law fluid [32], [33]. For power law fluids, the mass transfer coefficient is given by
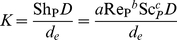
(3)where *a*, *b*, and *c* are empirical constants; *D* is the diffusion coefficient of dissolved xenon; *d_e_* = *εd_0_/(1−ε)* is the equivalent diameter of the hollow fiber bundle, with *d_0_* being the outer diameter of the fibers and *ε* being the void fraction of the gas-exchange module; and Sh_P_, Re_P_, and Sc_P_ are power law equivalents of the dimensionless Sherwood, Reynolds, and Schmidt numbers, respectively. Re_P_ and Sc_P_ can be expressed as
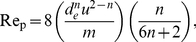
(4)and
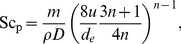
(5)where *ρ* is the density of blood, *u* is the average blood velocity in the module, *m* is the flow consistency index (essentially the power law analog of the Newtonian dynamic viscosity), and *n* is a dimensionless, empirical parameter known as the flow behavior index.

Before the magnetization reaching the arterial blood can be fully described, 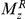, which is not constant in our work due to gas-phase relaxation, must be taken into account. Furthermore, the relaxation rate itself depends on the surface-to-volume ratio of the Tedlar reservoir and, therefore, changes over time due to deflation. Under steady-state gas flow conditions, we have shown that the time-dependent, gas-phase magnetization is well described by [34]

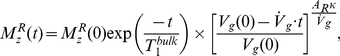
(6)where 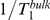 is the bulk, gas-phase (i.e., non-surface dependent) relaxation rate within the reservoir; *V_g_* is the reservoir volume; *A_R_* is the surface area of the reservoir; *k* is the surface relaxivity of Tedlar for ^129^Xe; and 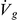 is the gas flow out of the reservoir. (Note, gas-phase relaxation within the transfer tubing and the module are negligible at gas flows exceeding ∼5 ml/min [16] and has therefore been omitted.) By substituting Eq. 6 into Eq. 2, the time-dependent, gas-phase signal resulting from EC infusion, *S_EC_*, becomes
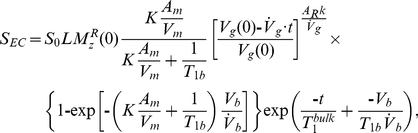
(7)where 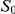 is a proportionality constant that accounts for factors such as the coil sensitivity, pulmonary perfusion, and diffusive gas exchange across the alveolar membrane, and *K* is obtained by combining Eqs. 3–5 with the literature values in Table 1.

## Results and Discussion

### Physiological Effects of HP ^129^Xe EC Infusion

In preliminary bench-top experiments, room temperature hetastarch was used to prime the EC circuit, but this sometimes led to severe reductions in body temperature and, occasionally, to the death of the animal (presumably due to cardiac arrest). However, both cardiac arrest and transient temperature reductions could avoided by heating the priming fluid to ∼37°C before initiating EC infusion. Temperature reductions were further mitigated by covering the EC circuit with a heating jacket (see Materials and Methods: Animal Preparation). After prolonged infusion, pulse distention decreased in most animals but was restored to baseline values by administering periodic, 1-ml injections of hetastarch.

Additionally, the blood oxygenation of some individuals in the bench-top studies dropped below 95% after prolonged EC infusion, presumably due to the removal of O_2_ from the blood. Increasing the O_2_ concentration in the breathing gas mixture to 35%, however, avoided decreases in O_2_ saturation. Although substantially increasing the O_2_ concentration of the breathing mixture above normoxic conditions (i.e., to values near 100%) has been shown to increase shunt fraction in rats due to absorption atelectasis [35], this effect is substantially reduced in less hyperoxic breathing mixtures [36]. Because the breathing mixture was only moderately hyperoxic, at most, minor effects on the ventilation/perfusion distribution were expected in this work.

In our final protocol, rats tolerated EC circulation at flows of up to 18.3 ml/min (i.e., 20–30% of cardiac output for a pentobarbital-anesthetized, 290–420-g rats [37]) for up to 4 hours without obvious adverse effects. In particular, heart rate did not deviate from baseline values during EC infusion. A concise summary of approaches used to optimize EC infusion MRI is provided as Table S1.

### Magnetization Dynamics

**Figure 2:**
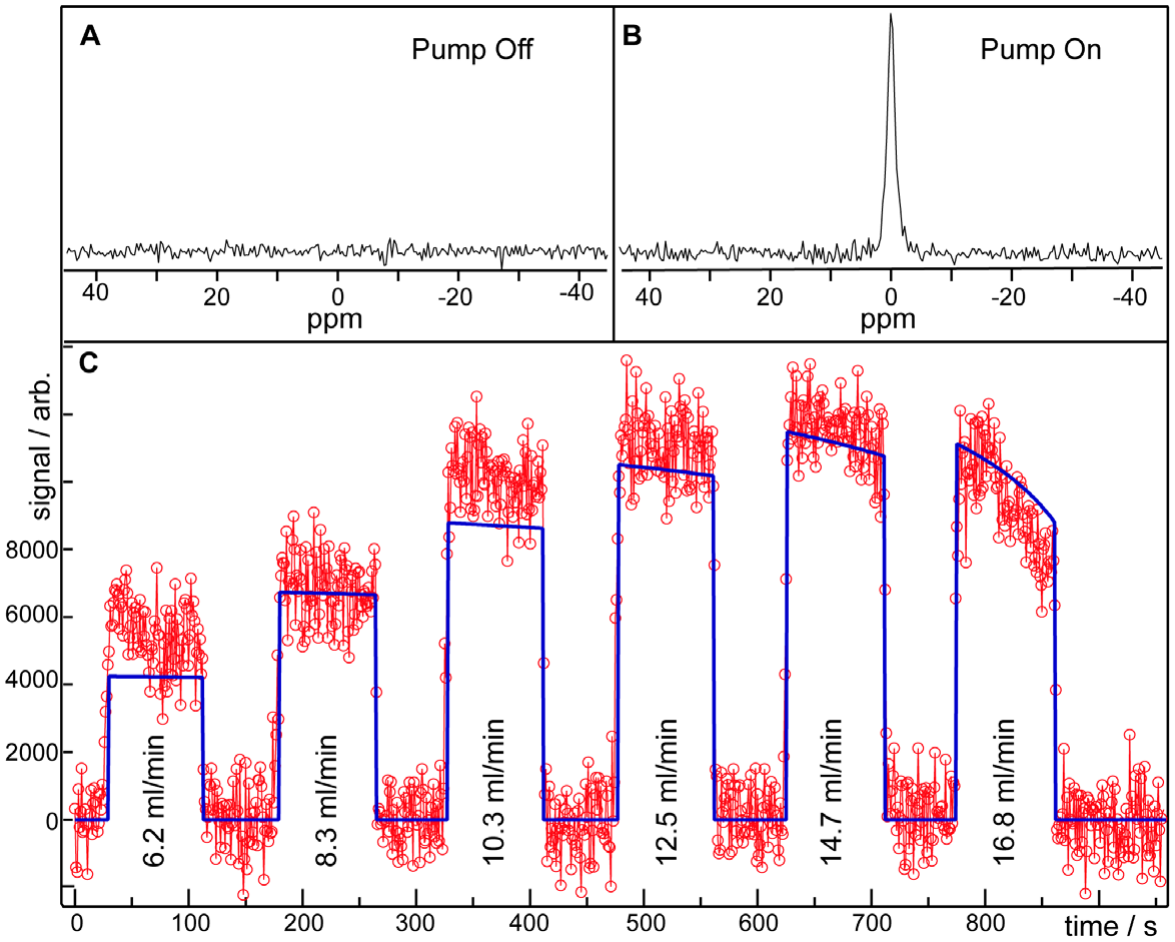
Signal dynamics during EC infusion. ^129^Xe gas flow through the exchange module was maintained at 10 ml/min throughout the experiment. **A.** Spectrum acquired at the frequency of ^129^Xe gas in the absence of EC flow. **B.** ^129^Xe spectrum obtained at an EC blood flow of 14.7 ml/min. **C.** Longtime ^129^Xe signal dynamics. The red circles depict the gaseous ^129^Xe signal as a function of time and blood flow through the peristaltic pump. Regions of near-zero signal intensity correspond to periods during which EC flow was suspended. The blue line is a fit of the data to Eq. 7. Regions of zero-flow and 2–5 s transitional periods between turning the pump on and off were omitted in the fit.

To validate our analytical model, the signal intensity of gaseous HP ^129^Xe reaching the alveolar spaces was investigated as a function of blood flow through the EC circuit. In these experiments, gas-phase ^129^Xe was selectively excited once per breath with a series of 90° RF pulses. At the beginning of these experiments, the peristaltic pump used to drive blood flow through the EC circuit was turned off, and no HP ^129^Xe signal was observed from the lungs (Fig. 2A). The pump was then repeatedly started (Fig. 2B) at increasingly high blood flow rates for periods of 1.5 min and stopped for periods of 1.0 min. In each case, once the pump was activated, HP ^129^Xe signal was detected within 2–5 s, depending on EC flow (Fig. 2C). Similarly, the gaseous signal decayed to zero within several seconds when the pump was turned off.

At the initial stages of EC infusion, signal intensity increased as flow through the EC circuit was increased. However, after 11–12 min of EC infusion, the signal intensity began to decay. While a portion of this decay was due to reduced mass transport efficiency [38], the majority of the signal decay was caused by longitudinal relaxation within the Tedlar HP ^129^Xe reservoir [16], [34]. Moreover, this reservoir relaxation depends on the surface-to-volume ratio of the Tedlar bag (see Eq. 6), and thus, the relaxation rate increased throughout the course of the experiment as the bag collapsed. The effect of this variable relaxation is most notably seen in the rapid signal drop-off observed in the data collected after ∼13 min of EC infusion flow (i.e., in the last half of the data collected at 16.8 ml/min). In longer experiments using a single, 300-ml bag of HP ^129^Xe (data not shown), detectable signal could be observed for more than 30 min, although with progressively reduced intensity.

Fig. 2C also shows a fit of the signal intensity data to Eq. 7 of our analytical model, using the literature values in Table 1. The scaling factor, 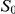, and the initial volume of the HP ^129^Xe reservoir, which was difficult to fill precisely, were used as the only free fitting parameters. Despite several uncertainties in the literature values (e.g., in several cases, literature values from bovine or human blood were used, because they have not been reported for rat blood), the fit adequately described the observed data. Moreover, the fit was particularly good at higher EC flow rates, which yielded the greatest signal intensity.

**Figure 3:**
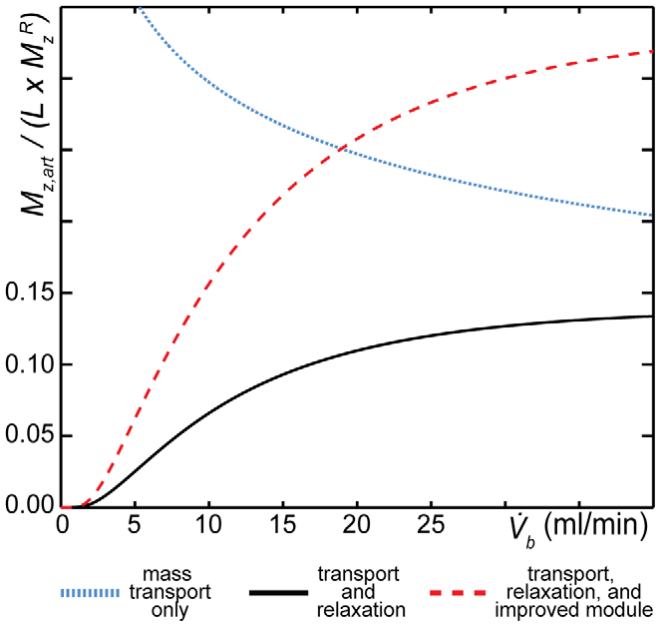
Simulated magnetization dynamics. Dissolved magnetization, in the absence of reservoir relaxation, is expressed as a fraction of its maximum possible value (i.e., the product of the gas-phase magnetization and the Ostwald solubility of xenon in blood). The dotted blue curve assumes the physical characteristics of the module used in this work but neglects dissolved-phase relaxation and thus represents only mass transport across the membranes. The solid black curve includes relaxation in the blood. The dashed red curve also accounts for relaxation in the blood but assumes that the module has been improved by doubling the membrane surface area to 200 cm^2^.

Although Fig. 2 provides insights into the signal dynamics during EC infusion and indicates that higher flows will yield higher signal intensities, relaxation-induced reductions in the magnetization arriving from the reservoir make empirically determining the optimal EC flow challenging. Fortunately, the quality of the fit in Fig. 2C suggests that our analytical model can also be used to simulate the expected magnetization dynamics in the absence of gas-phase relaxation within the HP ^129^Xe reservoir. Fig. 3 displays simulations of the flow-dependent arterial magnetization as a fraction of its maximum possible value, which is the product of the gas-phase magnetization reaching the exchange module and the Ostwald solubility of xenon.

The dotted blue line in Fig. 3 shows magnetization transport in the absence of relaxation and indicates that mass transport into the blood grows progressively less efficient as the blood flow is increased. For instance, assuming a solubility of *L* = 0.165 and a blood flow of 15 ml/min, only about 5% of the gas-phase xenon flowing through the module is taken by the blood. However, when ^129^Xe relaxation within the blood is included in the simulation (Fig. 3, solid black line), it is clear that the magnetization transport steadily increases with flow before reaching a plateau. A maximum value for the arterial magnetization (13.7% of 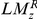) is predicted at a flow of ∼60 ml/min (not shown). However, achieving this maximum is not practical, because it would require a flow that is comparable to the entire cardiac output of 300–400 g rats [37]. Furthermore, this high flow rate would provide only a marginal improvement over the magnetization transport observed at a physiologically tolerable EC flow of 15 ml/min (10% of 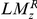).

Our analytical model also suggests that substantial improvements in magnetization transfer should be possible by modifying the design of the gas-exchange module. For instance, if the gas-exchange membrane surface area were increased from 100 to 200 cm^2^ (Fig. 3, dashed red line), the magnetization observed at an EC flow of 15 ml/min is expected to more than double due to improved mass transport. Similarly, reducing longitudinal relaxation within the exchange module is expected to increase the HP ^129^Xe magnetization delivered during EC infusion. For instance, relaxation losses would be diminished by ∼50% if the 0.6 ml volume within the module that contained no gas-exchange membranes were removed. Although this volume could not be completely eliminated, substantial qualitative signal intensity improvements were observed *in vivo* after this membrane-free volume was filled with 0.5-mm, siliconized glass beads (see Materials and Methods: HP ^129^Xe Accumulation and Infusion).

### Gas-Phase Signal Dynamics within a Breath

In small animal MRI using HP gases, data must be collected over multiple breaths to acquire a single image [10], [39], [40]. It is therefore vital to understand the HP gas signal variability during each breath to optimize the image acquisition strategy. Moreover, in the case of EC infusion, the temporal dynamics of HP ^129^Xe delivery to the alveolar spaces result directly from the underlying pulmonary physiology, and are thus of intrinsic interest. To investigate these dynamics over the entire breath cycle, animals were mechanically ventilated with a 250-ms inspiratory period, a 200-ms breath-hold at full tidal volume, and 550-ms period of passive exhalation. The signal intensity resulting from fresh gas-phase HP ^129^Xe magnetization diffusing from the capillary blood into the alveolar spaces was observed using a series of evenly spaced, selective, 90° RF pulses. Following each data acquisition, crusher gradients were applied to dephase any residual transverse magnetization.

**Figure 4:**
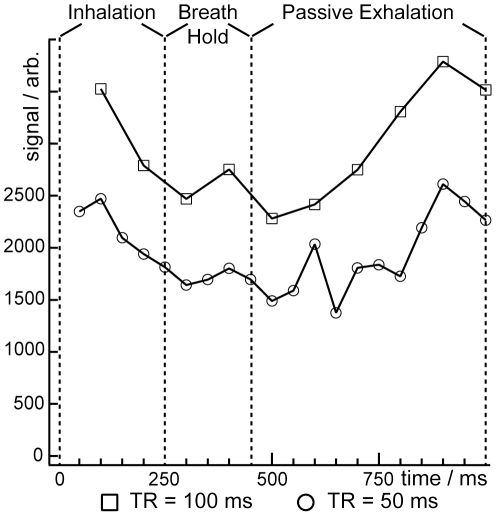
Signal dynamics during the breath cycle. Data are the gaseous HP ^129^Xe signal intensity observed from two different animals using 50-ms (circles) and 100-ms (squares) repetition times between 90° RF pulses. Lines are intended only as guides.

Although any influence from residual and longitudinal and transverse magnetization was removed in these experiments, additional factors also affect HP ^129^Xe delivery, and these must be accounted for if the signal dynamics within the breath cycle are to be evaluated. For instance, a repetition time of TR = 1 s (i.e., 1 RF pulse per breath) was used to obtained the data shown in Fig. 2, but significantly shorter TRs are needed to study the signal dynamics within a single breath. However, Eq. 1 predicts that shorter TRs will lead to substantial reductions in HP ^129^Xe signal intensity. To overcome low SNR and, incidentally, to reduce the influence of any cardiac cycle-induced signal fluctuations, spectra were acquired over 50 breaths, and the individual spectra obtained at each time-point within the breath cycle were averaged. The resulting signal dynamics data obtained from two different animals are shown in Fig. 4.

The most obvious feature in Fig. 4 is that signal intensity observed with a TR of 100 ms was ∼50% larger than that seen with a TR of 50 ms. While the two datasets cannot be quantitatively compared because they were collected from different animals, the trend is in agreement with Eq. 1, which predicts increased HP ^129^Xe accumulation within the alveolar spaces at longer TRs. However, for both animals, the signal intensity varied substantially throughout the breath cycle (e.g., the standard deviation of the signal is 18.0% of the mean in both cases). More specifically, using a 50-ms TR, the signal intensity decreased by 41% near peak inspiration before returning to higher values at the end of the breath. A similar decrease of 35% near peak inspiration followed by a subsequent increase was observed for the 100-ms TR data.

Interestingly, the times at which the lowest signal is observed corresponds to the breath-hold and the periods immediately before and immediately following the breath-hold. That is, the lowest flux of HP ^129^Xe into the alveolar spaces occurred during the portion of the breath cycle at which the alveolar pressure and volume was the highest. This observation of low alveolar magnetization at high alveolar volumes is in agreement with the predictions of Eq. 1, which, using 90° RF pulses at short TRs, becomes 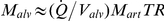. Additionally, these signal dynamics may also be influenced by variability in the capillary blood volume, which Weibel *et al.* [41] reported to decrease as a result of positive pressure inflation.

The above observations hint at several pathways for optimizing MR acquisitions within a given breath cycle to yield higher SNR. In agreement with Eq. 1, acquiring data using a longer TR is expected to yield higher signal intensities as would imaging near end-expiration. Moreover, the 200-ms breath-hold period used to acquire the spectroscopy data in Fig. 4 should be eliminated to increase the overall flux of HP ^129^Xe into the alveolar spaces. Also, removing the extended breath-hold will provide prolonged, motion-free periods during which imaging can be performed at end-expiration.

### Gas-Phase MR Imaging

**Figure 5:**
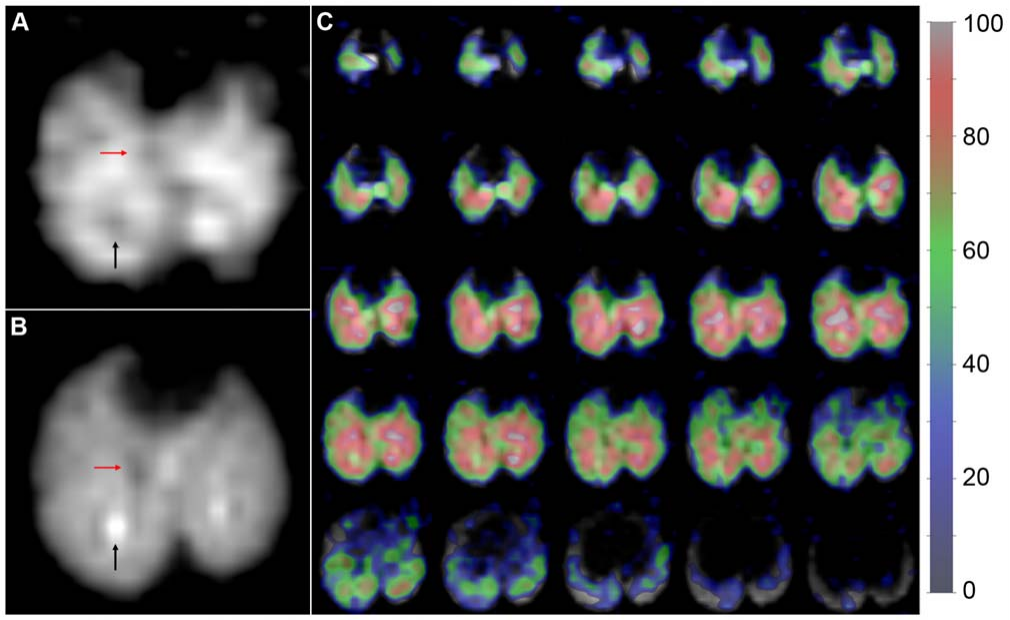
Gas-phase ^129^Xe MR imaging. **A.** Axial slice from an EC infusion image (EC flow ∼15 ml/min) acquired with a nominal isotropic resolution of 2×2×2 mm^3^. Black arrow indicates a signal void in the image corresponding to the location of a major airway. Red arrow indicates a hypointense region that likely corresponds to a large blood vessel. **B.** Axial slice from the corresponding ventilation image acquired with 1×1×2 mm^3^ nominal resolution. Black arrow indicates a large airway in the same location as the signal void in A (also marked by a black arrow). Red arrow points to a hypointense region corresponding to the red arrow in B. **C.** Complete 3D, gas-phase, EC infusion image (color) overlaid on the 3D ventilation image (grayscale). The signal intensity of the EC infusion image, in arbitrary units, is indicated in the legend.

Using an EC flow of ∼15 ml/min and TR = 180 ms, 3D images of ^129^Xe gas were acquired at end-expiration with a nominal resolution of 2×2×2 mm^3^. The central axial slice from a representative image is shown in Fig. 5A. The SNR of this slice is 9.6, and the average SNR for the entire image is 9.2, with the SNR of the slices ranging from 6 to 11. Similar signal intensity was observed for all three 3D EC infusion images acquired in this work (mean SNR = 9.2±0.6). The corresponding 3D, HP ^129^Xe ventilation image was acquired with 1×1×2 mm^3^ resolution and is shown in Fig. 5B.

Although the infusion image in Fig. 5 was acquired at a lower spatial resolution than the corresponding ventilation image, it displays many of the same spatial features as the corresponding ventilation image. For instance, the ventilation image in Fig. 5B displays a region of low signal intensity, likely corresponding to the location of a large blood vessel, that is matched by a similar hypointense region in the corresponding EC infusion image in Fig. 5A (see red arrows). The similarities are better appreciated in Fig. 5C, which displays the entire, 3D EC infusion image overlaid in color on the grayscale ventilation image and indicates that diffusive gas exchange occurred throughout much of the ventilated portions of the lungs. The spatial agreement within the lung parenchyma between the two types of gas-phase ^129^Xe images is not surprising, because diffusion across the alveolar membrane is expected to be unobstructed in healthy animals. Furthermore, the signal intensity of the EC infusion image scales with perfusion (see Eq. 1), and ventilation and perfusion are expected to be well matched in healthy animals, with the ventilation-perfusion ratio being 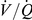
≈1 [42].

However, some exceptions to this general agreement are also observed. For example, larger airways display high signal intensity in the inhaled ^129^Xe images (e.g., see black arrow, Fig. 5B), but essentially no signal is seen in the corresponding portions of the EC infusion images (black arrow, Fig. 5A). This discrepancy is consistent with observations made using injected HP ^129^Xe [14], and is logical, because no diffusive gas elimination occurs in larger airways.

Under appropriate experimental conditions (i.e., using 90° pulses with short, fixed TRs), the HP ^129^Xe magnetization reaching the alveoli due to EC infusion will be completely consumed within the gas-exchange region, and the resulting MR signal will be directly proportional to pulmonary perfusion (see Eq. 1). Thus, it should be possible to generate normalized 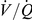 images, similar to those obtained with nuclear imaging modalities [43]. Moreover, the HP ^129^Xe images in Fig. 5 have a spatial resolution comparable to that currently achieved in small animal lungs with Single Photon Emission Tomography (SPECT) [44] and Positron Emission Tomography (PET) [45]. However, these EC infusion images were acquired within ∼20 min, representing a 4–5-fold advantage in temporal resolution over nuclear imaging. Further, unlike many nuclear imaging tracers, which require days for complete clearance between scans [46], repeated imaging with ^129^Xe is limited only by the time needed for hyperpolarization (∼30 min). These characteristics, combined with the ability to almost instantaneously initiate or terminate ^129^Xe delivery, suggest that EC infusion may allow 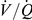 to be rapidly assessed in small animal models of pulmonary diseases that progress on a timescale of hours, such as acute lung injury [47].

### 3D Dissolved-Phase MR Imaging

**Figure 6:**
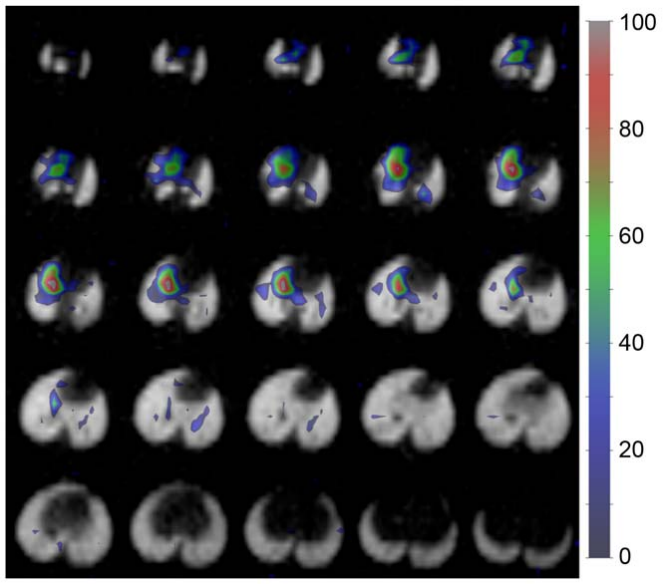
Dissolved ^129^Xe MR imaging. Axial, EC ^129^Xe infusion image (color; 2×2×2 mm^3^ nominal resolution; EC flow ∼15 ml/min) overlaid on the corresponding ventilation image (grayscale; 1×1×2 mm^3^ nominal resolution). High signal intensity is seen from the right heart and pulmonary arteries, but not from the lung parenchyma, due to RF attenuation and rapid diffusive losses of ^129^Xe from the capillary blood into the alveolar gas spaces. The signal intensity of the dissolved image (arbitrary units) is indicated in the legend.

To investigate the extent to which EC infusion will enable ^129^Xe MRI of thoracic structures other than the lungs, two rats were also imaged by selectively exciting dissolved ^129^Xe. An example of a dissolved, HP ^129^Xe MR image acquired during EC infusion (2×2×2 mm^3^ nominal resolution) is shown in Fig. 6. This image displays the highest signal intensity (SNR = 8–11) in the right heart and pulmonary arteries, but essentially no signal is seen in the lung parenchyma. This distribution stands in stark contrast to previous dissolved ^129^Xe MR images of the thorax [10], [11], [12], which were obtained by inhaling ^129^Xe and allowing the gas to dissolve in the pulmonary tissues. Once inhaled, rapid gas exchange then replenishes the dissolved magnetization on a timescale of tens of milliseconds [10], [48], and thus enables substantial signal averaging and yields reasonably high signal from the lung parenchyma. For inhaled ^129^Xe, the RF pulses used for imaging rapidly attenuate the HP magnetization as it flows away from the gas-exchange tissues and, as a result, only weak signal was observed in the heart [11]. However, during EC infusion, the magnetization dynamics within the lungs are substantially different.

Unlike when magnetization replenishment occurs via diffusive gas uptake, dissolved magnetization replenishment during EC infusion depends on blood flow from the heart, which occurs on the timescale of seconds and is attenuated by RF prior to reaching the parenchyma. More importantly, because xenon is only moderately soluble in pulmonary tissues, HP ^129^Xe rapidly diffuses into the alveolar spaces [15], leaving only a small equilibrium percentage (∼5%) of the initial arterial magnetization remaining in the gas-exchange region. Therefore, little signal intensity is observed in the lung parenchyma, and the small regions that are observed likely correspond to the larger blood vessels.

While this initial study focused on imaging the lungs and adjacent structures, the ability to image dissolved ^129^Xe in the larger blood vessels and the heart suggest that EC infusion may represent a general method for delivering HP ^129^Xe to the bloodstream. That is, simply by changing vascular route for HP ^129^Xe infusion, it should be possible to deliver HP ^129^Xe to essentially any organ in the body. In doing so, a wide variety of novel applications, including higher resolution ^129^Xe imaging of the brain [49] will be possible. Another potential application of this technique is ^129^Xe-based molecular imaging using cryptophane biosensors [50], [51], [52], which have shown promise in proof-of-principle, *in vitro* studies. As a final point, the EC circuit used in this work was adopted specifically to accommodate the relatively small size of rats. Another, less invasive method would use an infusion catheter, similar to respiratory assist catheters already in development [53], to deliver HP ^129^Xe to the blood of larger animals.

### Conclusions

Here, we have extended earlier *in vitro* work with hydrophobic, hollow-fiber gas-exchange modules and demonstrated that this technology can deliver HP ^129^Xe directly to the bloodstream of live animals. Moreover, EC infusion can be continued, albeit with relaxational losses in signal intensity, for periods of 30 minutes or more using only 300 ml of xenon gas. Once delivered to the venous circuit, HP ^129^Xe arrives in the alveolar airspace by a combination of diffusive gas exchange and pulmonary perfusion. Thus, gas-phase ^129^Xe MR images of the lungs obtained during EC infusion reflect the 3D spatial distribution of the underlying gas-elimination processes. By comparing these images with the corresponding 3D ventilation images, it may be possible to examine variations in the pulmonary ventilation-perfusion ratio with spatial resolution comparable to or better than that of traditional nuclear imaging modalities, but with substantial improvements in temporal resolution. Furthermore, unlike the radioactive contrast agents used in SPECT imaging of pulmonary perfusion, the signal from HP ^129^Xe delivered by EC infusion can be modulated with RF pulses and turned on and off at will simply by controlling blood flow through the EC circuit. Together, these properties promise to enable novel studies of rapidly changing pulmonary physiology in small animals.

We have also adapted our previously published analytical model describing the signal dynamics of membrane-infused HP ^129^Xe so that it accounts for variability in the source magnetization and the non-Newtonian behavior of blood. Of practical importance, the model predicts that the use of high EC flows (i.e., >20 ml/min), which may not be tolerated well by live rats, will yield only modest increases in HP ^129^Xe signal intensity over more moderate EC flows of ∼15 ml/min that we used for imaging. Additionally, this model predicts that it should be possible to design improved exchange modules that will support the delivery of significantly higher levels of HP ^129^Xe magnetization to the lungs.

Finally, we have shown that EC infusion generates substantial dissolved-phase ^129^Xe signal in regions that are distal to the gas-exchange tissues. This suggests that, by using different vascular points of entry for the EC circuit, it should be possible to deliver HP ^129^Xe to essentially any organ in the body. In doing so, EC infusion promises to make possible a wide range of functional and molecular imaging approaches that are currently not feasible using inhaled ^129^Xe.

## Supporting Information

Table S1 **Extracorporeal Infusion Optimization Strategies.** ^A^ Observed during bench top experiments prior to EC infusion MR experiments.
(DOC)
