# Supplementary material for: In Vivo MR Imaging of Pulmonary Perfusion and Gas Exchange in Rats via Continuous Extracorporeal Infusion of Hyperpolarized 129Xe
Source: PLoS One. 2012 Feb 21;7(2):e31306. doi: 10.1371/journal.pone.0031306 (PMC3283644; doi:10.1371/journal.pone.0031306)
Supplement: Table S1 — Extracorporeal Infusion Optimization Strategies. A Observed during bench top experiments prior to EC infusion MR experiments. (DOC) [file pone.0031306.s001.doc]

**Table S1.** Extracorporeal Infusion Optimization Strategies

| **Experimental Challenge** | **Optimization Strategy** |
| --- | --- |
| EC infusion Diminished O_2_ saturation **^A^** | Increase O_2_ concentration to 35% in breathing gas mixture. |
| EC infusion lowered body temperature **^A^** | Heating EC circuit priming fluid, gas exchange module, and blood transfer lines |
| Decreased peripheral, arterial pressure during prolonged EC infusion **^A^** | Periodic injections of non-crystalloid hetastatch solution |
| Substantial dissolved HP ^129^Xe T_1_ relaxation within the exchange module | Empty volume in the gas exchange module was filled silicone-treated glass beads |
| Limited time for gaseous ^129^Xe to accumulated in the alveolar spaces | EC infusion MRI with increased TR to allow greater alveolar ^129^Xe accumulation |
| Long TRs allowed limited number of views to be acquired during a breath-hold | Imaging was performed during longer, motion free period at end-expiration |
| Short T_2_^*^ for dissolved-phase ^129^Xe | Bandwidth was increased for dissolved, EC infusion MRI |
